# Genome dynamics mediated by repetitive and mobile elements in *Xanthomonas citri* pv. *durantae*

**DOI:** 10.1099/acmi.0.000415

**Published:** 2022-10-03

**Authors:** Rekha Rana, Kanika Bansal, Amandeep Kaur, Prabhu B. Patil

**Affiliations:** ^1^​ Bacterial Genomics and Evolution Laboratory, CSIR-Institute of Microbial Technology, Chandigarh, India; ^2^​ Academy of Scientific and Innovative Research (AcSIR), Ghaziabad, Uttar Pradesh, India

**Keywords:** comparative genomics, integrative and conjugative elements, IS elements, pathovar, transcription activator-like effectors (TALEs), *Xanthomonas citri* pv. *durantae*

## Abstract

*
Xanthomonas* is a highly evolved group of phytopathogenic bacteria infecting nearly 400 host plants having vast genomic resources available with heterogenicity in representation from different species and pathovars. Unfortunately, the wealth of data is extremely biased and restricted to a few *Xanthomonas* pathogens that infect economically important plants, while those reported to infect the most diverse plants remain neglected. In the present study, we report the first complete genome sequence of *Xanthomonas citri* pv. *durantae* that was reported to infect *Duranta repens L*. or golden dewdrop, a hedge plant of ornamental importance native to the American region. Phylogenomic analysis with its closest relatives placed it amongst *X. citri* pv. *citri* A* pathotype strains and further comparative studies revealed various large unique genomic regions of chromosomal origin. The association of integrative and conjugative elements and prophages with unique genomic regions suggests the role of mobilome in genome dynamics. A large number of IS elements and transcription activator-like effectors encoding genes on each of the four plasmids indicate the further scope of diversification in *Xanthomonas *.

## Introduction

*Xanthomonas* is a complex genus of Gram-negative phytopathogenic bacteria comprising more than 34 species (LPSN, http://www.bacterio.net, accessed on 5 August 2022) [1] capable of infecting around 400 plants [2–4]. It is an extensively studied group of phytopathogens with a plethora of genomic data available in the databases. However, there is a lack of complete genomic information of *Xanthomonas* pathogens reported to infect diverse hosts. *Xanthomonas citri* pv. *durantae* (Xdur) infects *Duranta repens L*. (golden dewdrop), a flowering plant of the Verbenaceae family native to the American region, popularly grown as hedge across the globe (www.cabi.org). *Duranta repens* is widespread in Mexico, the Caribbean, and most of South America, as well as in Florida, California, and Texas in the United States of America; it serves its ornamental importance as a garden plant and windbreaks [5]. Disease symptoms involve the extensive formation of enlarged, angular spots with a brownish centre and slightly raised margins [6, 7]. The first report of *Xanthomonas* infecting *Duranta repens* was from India in 1957 [6, 7]. In 2013, an unidentified strain of the *Xanthomonas* genus was reported to cause symptoms comparable to Xdur infection on golden dewdrop in Florida, USA [8].

Previous studies based on marker genes and phylo-taxonogenomic analyses, including average nucleotide identity and digital DNA–DNA hybridization values, suggested *Xanthomonas citri* pv. *citri* (Xcc), which causes citrus bacterial canker (CBC) and *Xanthomonas citri* pv. *durantae* (Xdur) are closely related [9, 10], and Xdur is one of the constituent pathovars of *X. citri* [11, 12]. As these earlier studies were based on draft genome sequences, mechanistic details of genome dynamics in evolution and variation of closely related pathovars were lacking. The short-read assemblies make it challenging to study genes of repetitive nature, such as transcription activator-like effectors (TALEs), which are crucial determinants of pathogenicity [13] and IS (Insertion Sequences) elements, which are small mobile genetic elements dispersed over a genome, responsible for genome plasticity as well as genomic rearrangements [14]. The emergence of third-generation sequencing technologies has created immense opportunities for investigating the role of mobile genetic elements and repetitive elements in host diversification with much more precision [15].

The present study reports the first complete genome-based investigation of *Xanthomonas citri* pv. *durantae* strain LMG696, which is available in the culture collections and the NCBI database (https://www.ncbi.nlm.nih.gov/assembly/GCF_019201325.1/) as *X. campestris* pv. *durantae* LMG696. LMG696 is the reference strain of the pathovar. Comprehensive genome comparisons with the complete genomes of *X. citri* pv. *citri* strains led to identifying five large dynamic regions associated with integrative and conjugative elements and prophages in the chromosome. Our study also observed variations in IS elements and TALE repertoire during the diversification of genomes. This suggests the importance of complete genome-based studies of *Xanthomonas* strains reported to infect hosts of less economic importance and also investigating genome dynamics apart from phylogenomics.

## Methods

### Genome sequencing, annotation and assembly

Xdur LMG696 procured from BCCM (Belgium Coordinated Collections of Microorganisms) was grown overnight in nutrient broth at 28 °C. Cells were harvested, and genomic DNA was isolated using the Quick-DNA Fungal/Bacterial Miniprep Kit (Zymo Research, Irvine, CA). Quantitative and qualitative analysis of genomic DNA was carried out using Qubit 4 fluorometer (Invitrogen; Thermo Fisher Scientific) and Nanodrop 1000 (Thermo Fisher Scientific). A ligation sequencing kit, SQK-LSK109 (Oxford Nanopore Technologies), was used for library preparation. All bead washing steps were performed using AMPure beads (Beckman Coulter). Native barcoding and adaptor ligation steps were performed as per the protocol given by Oxford Nanopore Technologies using the Native barcoding kit (EXP-NBD104). Finally, 12 µl of prepared DNA library was sequenced using MinION R10 flow cell (FLO-MIN110) with MinKNOW software v3.6.5 for 72 h (http://community.nanoporetech.com; Oxford Nanopore Technologies). Nanopore FAST5 raw reads were base called and converted to the FASTQ format using guppy v4.2.2+effbof8 software (http://community.nanoporetech.com). Reads obtained were filtered using Filtlong v0.20 to obtain reads with length ≥5 000 bp (--min_length 5 000) and discarded worst 10 % of the reads (--keep_percent 90) until 500 Mbp remained for assembly (--target_bases 500 000 000) (https://github.com/rrwick/Filtlong). Hybrid assembly of filtered ONT reads with previously sequenced Illumina reads was done using Unicycler v0.4.8 with the default mode [16]. Assembled genome was then polished for multiple rounds using Pilon v1.22 [17] with Illumina short reads. The genome was evaluated for completeness using CheckM v1.0.13 [18], and average genome coverage was calculated using BBMap v38.42 [19]. The genome was submitted to the NCBI Whole Genome Shotgun (WGS) portal and annotated using the NCBI’s (https://www.ncbi.nlm.nih.gov/genome/annotation_prok/) Prokaryotic Genome Annotation Pipeline (PGAP). Pictorial representation of the complete genome of LMG696 and plasmids was drawn using CGview comparison tool (CCT) [20] and CGView Server ^BETA^ [21], respectively.

### Phylogeny construction and comparative genomics

For the phylogenetic analysis, 35 available complete genomes of *X. citri* pv. *citri* were retrieved from NCBI accessed on 15 May 2021 (https://www.ncbi.nlm.nih.gov/). The genomes were annotated with Prokka v1.14.6 [22], and Roary v3.13.0 [23] was used to obtain core gene alignment. The core gene alignment file was used as an input for PhyML v20160207 [24] to generate an initial tree. Further, recombination sites were detected using ClonalFrameML v1.12 [25] and masked with maskrc-svg (https://github.com/kwongj/maskrc-svg). The final non-recombining phylogenetic tree was constructed using PhyML v20160207 [24] and visualized with iTOL v6.1.2 [26]. *X. citri* pv. *glycines* CFBP2526 was used as an outgroup. Genome comparison analysis was carried out using BRIG v0.95 [27]. Integrative and conjugative elements (ICEs) and prophages were predicted with the help of ICEfinder [28] and PHASTER [29], respectively.

### IS elements and transcription activator-like effectors (TALEs)

IS elements were identified using ISsaga 2.0 web server [30]. The TALEs in the Xcc strains and Xdur LMG696 were identified by the ‘TALE Prediction’ tool of AnnoTALE software version 1.5 [31]. Further, identified TALEs were assigned to different classes by the ‘TALE class assignment’ tool of AnnoTALE. A neighbor-joining tree of the central repeat regions, including RVDs of the TALEs, was constructed using the DisTAL v1.1 module of the QueTAL suite [32]. The DisTAL constructs phylogeny based on an alignment of central repeat regions of TALEs.

## Results and discussion

### Distinct genomic features of Xdur LMG696

**Fig. 1.**
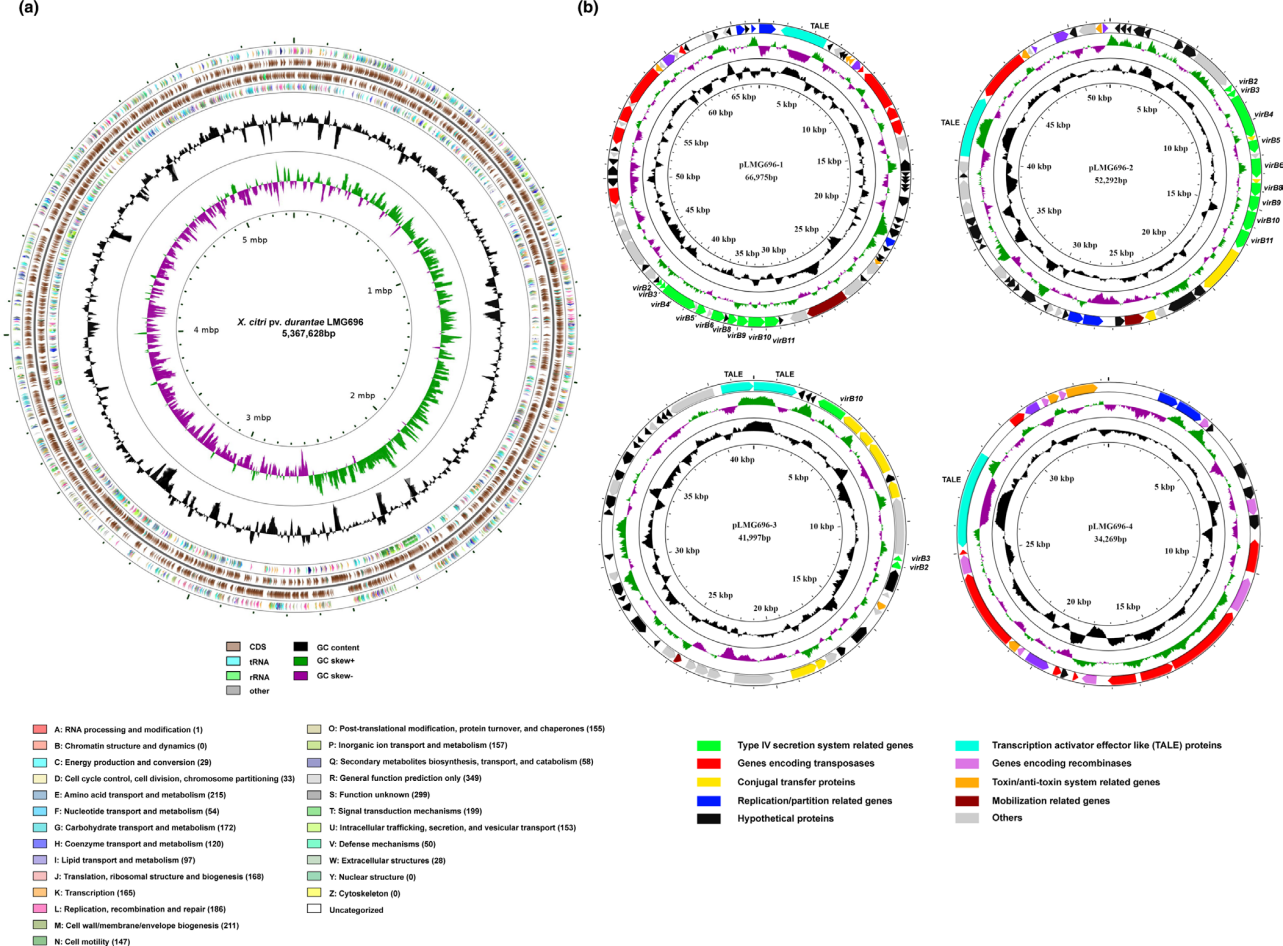
a) Circular map of *X. citri* pv. *durantae* LMG696. Starting from the outermost ring to centre: CDS genes on the forward strand coloured according to their COG classification, CDS genes and RNAs on the forward strand, CDS genes and RNAs on the reverse strand, CDS genes on the reverse strand coloured according to their COG classification, GC content, and GC skew. **b**) Pictorial representation of Xdur plasmids; pLMG696-1, pLMG696-2, pLMG696-3, and pLMG696-4. The outermost ring represents protein-coding genes; clockwise and anti-clockwise arrows indicate the forward and reverse orientation of the genes, respectively. Two innermost rings represent GC content (black) and +/-GC skew (purple and green). The circular scale gives genome coordinates. Type IV secretion system and TALEs are indicated with green and turquoise colours, respectively.

Xdur LMG696 harbours a circular chromosome of 5.36 Mb (Fig. 1a) and four plasmids (Fig. 1b) named pLMG696-1 (66.9 kb), pLMG696-2 (52.2 kb), pLMG696-3 (41.9 kb), pLMG696-4 (34.2 kb) having NCBI accession numbers CP066343, CP066344, CP066345, CP066346, and CP066347, respectively. The genome was 100 % complete with 131 x average genome coverage. The complete genome has 4753 coding sequences with 53 tRNAs and two copies of rRNA operons (5S, 16S, 23S). Out of 4533 coding sequences, 3046 were assigned to clusters of orthologous groups (COGs) categories using CCT (Fig. 1a) [20]. The major COG categories with the maximum number of CDSs were amino acid transport and metabolism (E), cell wall/membrane/envelope biogenesis (M), signal transduction mechanisms (T), and replication, recombination, and repair (L) (Fig. 1a). Overall the genome size of Xdur LMG696 is comparable to other *X. citri* pathovars indicating there is no major genome reduction or expansion associated with the evolution or emergence of this pathovar.

The plasmids carry genes encoding partition and replication proteins such as *parA, parB*, conjugal transfer protein-encoding genes such as *traI, traG, trbM, trbG, trbF, trbL*, proteins related to toxin/anti-toxin systems, mobilization proteins, transposases and a large number of hypothetical proteins. The type IV secretion system reported by Bansal and co-workers on contig 29 of the draft genome of LMG696 is located on plasmid pLMG696-1 (Fig. 1b) [9]. As reported earlier [12], this type IV secretion system is also associated with the plasmids of *X. citri* pv. *citri* strain TX160149, *X. campestris* pv. *campestris* CN18 and *X. campestris* pv. *campestris* CN03. Apart from this, pLMG696-2 also has a type IV secretion system cluster, which shares homology to *X. citri* pv. *citri* strain 29–1 and *X. citri* pv. *citri* strain LH276 (Fig. 1b). This indicates that plasmids carrying distinct type IV secretion systems might be playing a role in the evolution and adaptation of Xdur pathovar.

### Phylogenetic and genome comparison analysis of Xdur LMG696 with its closest relatives

**Fig. 2.**
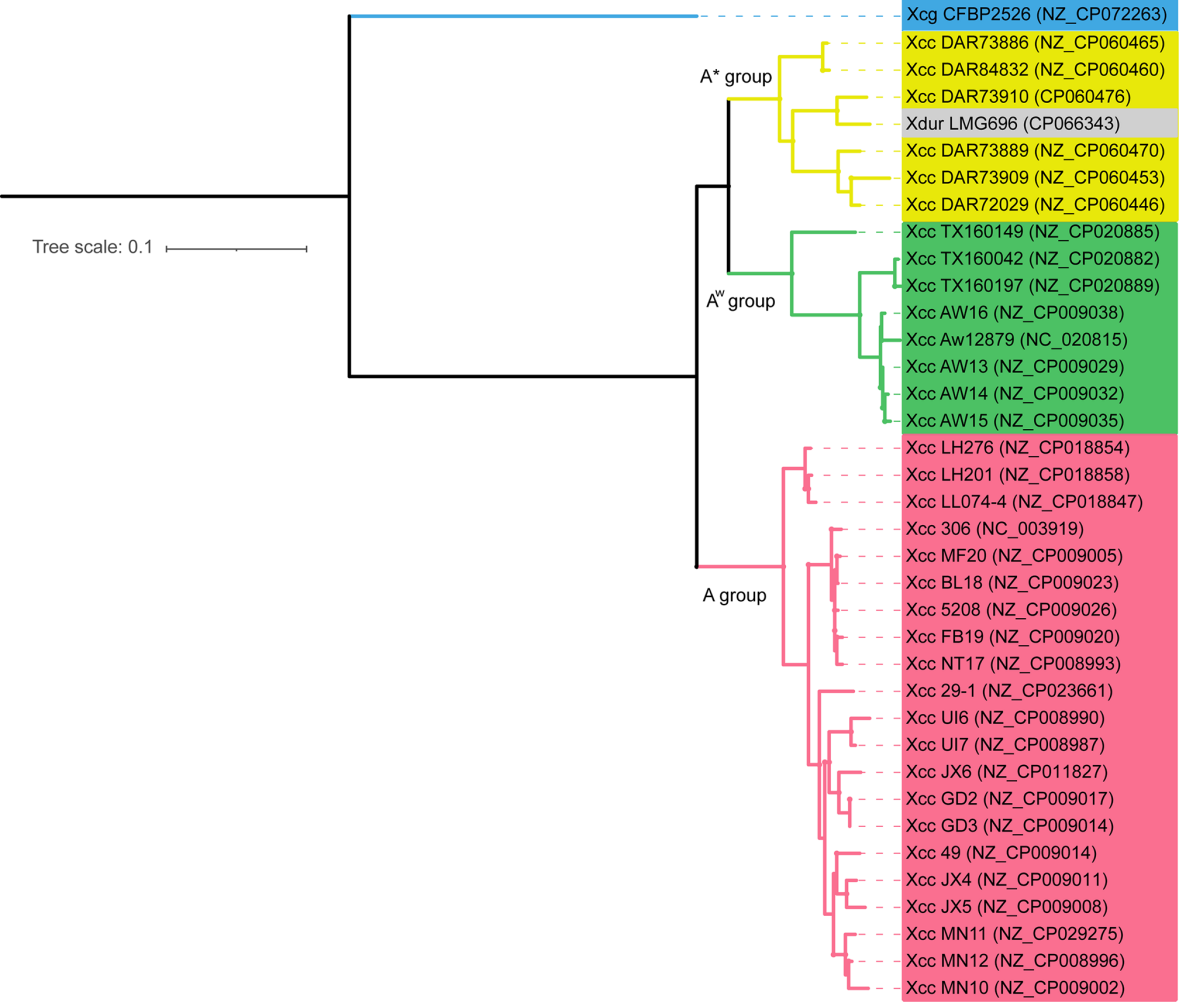
A maximum-likelihood phylogenetic tree of *X. citri* pv. *durantae* and 35 *X *. *citri* pv. *citri* strains based on core gene alignment constructed using PhyML [24]. Phylogenetic grouping is colour-coded according to Xcc pathotypes along with their NCBI accession numbers: yellow, A* pathotype; green, A^w^ pathotype; pink, A pathotype. Xdur LMG696 is represented in grey colour. *X. citri* pv. *glycines* CFBP2526 was used as an outgroup, here depicted in blue colour.

The phylogeny constructed using the complete genomes of *X. citri* pv. *citri* strains along with Xdur LMG696 was consistent with the previous studies [33], where the phylogenetic analysis revealed three main groups correlated with three *X *. *citri* pv. *citri* pathotypes; A, A* and A^w^. Interestingly, Xdur LMG696 was clustered within the A* group (Fig. 2). The three pathotypes of *X. citri* pv. *citri*, A, A* and A^w^ differ in their host range and the host plant defence responses towards them. Xcc A pathotype has a broad host range infecting almost all citrus plants, while A* and A^w^ restrict themselves to key lime (*Citrus aurantifolia*) and alemow (*Citrus macrophylla*) [12, 33]. A^w^ pathotype differs from the A* pathotype as it shows a hypersensitive response in grapefruit and sweet orange given to the presence of the *XopAG*/*avrGf1* gene [12, 33].

**Fig. 3.**
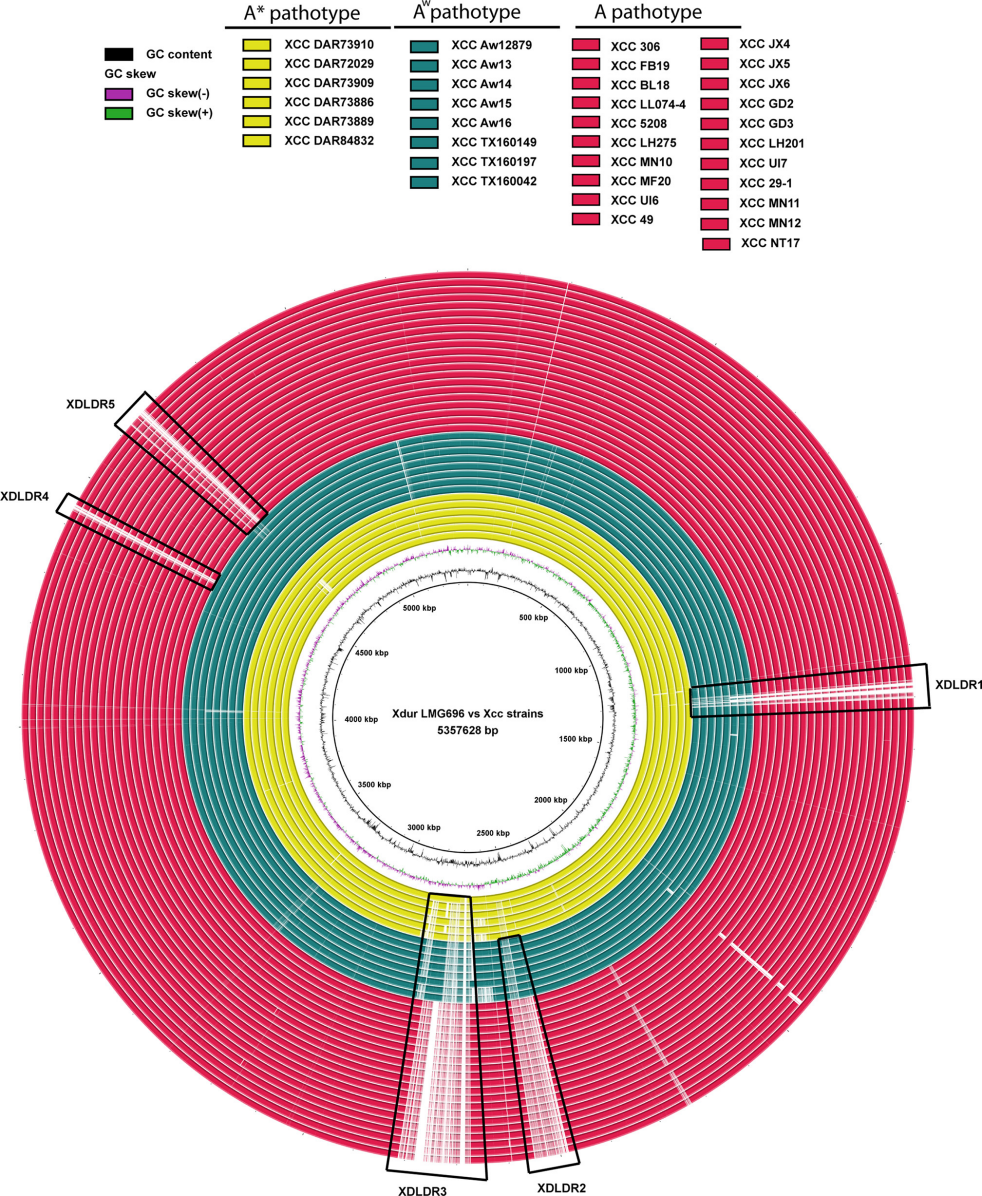
Comparative genome map of Xcc strains against Xdur LMG696 constructed using BRIG [27]. Concatenated rings from the inside out represent shared homology between Xdur LMG696 (reference genome) with ring 1 to 6 (yellow-coloured) Xcc A*pathotypes, ring 7–14 (green-coloured) Xcc Aw pathotypes, and ring 15–35 (red-coloured) Xcc A pathotypes with white-coloured area represents a genome lacking a particular region (highlighted in black boxes, XDLDR1-XDLDR5). Two innermost rings indicate GC content and GC skew, respectively.

Genome comparison analysis of Xdur LMG696 with these complete genome sequences of 35 *X *. *citri* pv. *citri* strains revealed five regions that are unique to Xdur LMG696, termed Xdur large dynamic regions (XDLDRs) (Fig. 3). XDLDR1, XDLDR4, and XDLDR5 were absent in A pathotype strains, while all strains of A* and A^w^ pathotypes have XDLDR4 and XDLDR5, with some of the A^w^ pathotype strains lacking XDLDR1 (Fig. 3). Further, PHASTER [29] analysis revealed the presence of a prophage in the XDLDR5 region. XDLDR2 was absent in both the A and A^w^ pathotypes, while a significant part of XDLDR3 was absent in all Xcc strains (Fig. 3). On further analysis, XDLDR2 and XDLDR3 were found to harbour ICE-related genes. ICEfinder [28] analysis revealed the presence of two ICE regions in the Xdur LMG696 genome. Interestingly, both of these ICEs were mapped to the XDLDRs. One of the ICEs (coordinates 2 483 602–2 550 615) was part of the XDLDR2 and another ICE (coordinates 2 624 779–2 743 795) was part of XDLDR3. These regions carried type IV secretion system-related genes such as *virB6*, *virB4*, *traI*, and *traD*. Interestingly, XDLDR3 was found to harbour a gene encoding heavy metal translocating P-type ATPase (Xdur_12075) and multidrug efflux pump-related genes (Xdur_12095, Xdur_12100, Xdur_12105) (Table S1, available in the online version of this article). These five regions carry a large number of IS elements and hypothetical genes. Apart from these, genes encoding AlpA family, LysR family, TetR family, and helix-turn-helix transcriptional regulators, DNA repair system proteins, DNA replication proteins, methyltransferases, ABC transporters, and proteins domains of unknown function were common in the XDLDRs (Table S1).

### Comparison of repetitive elements

**Fig. 4.**
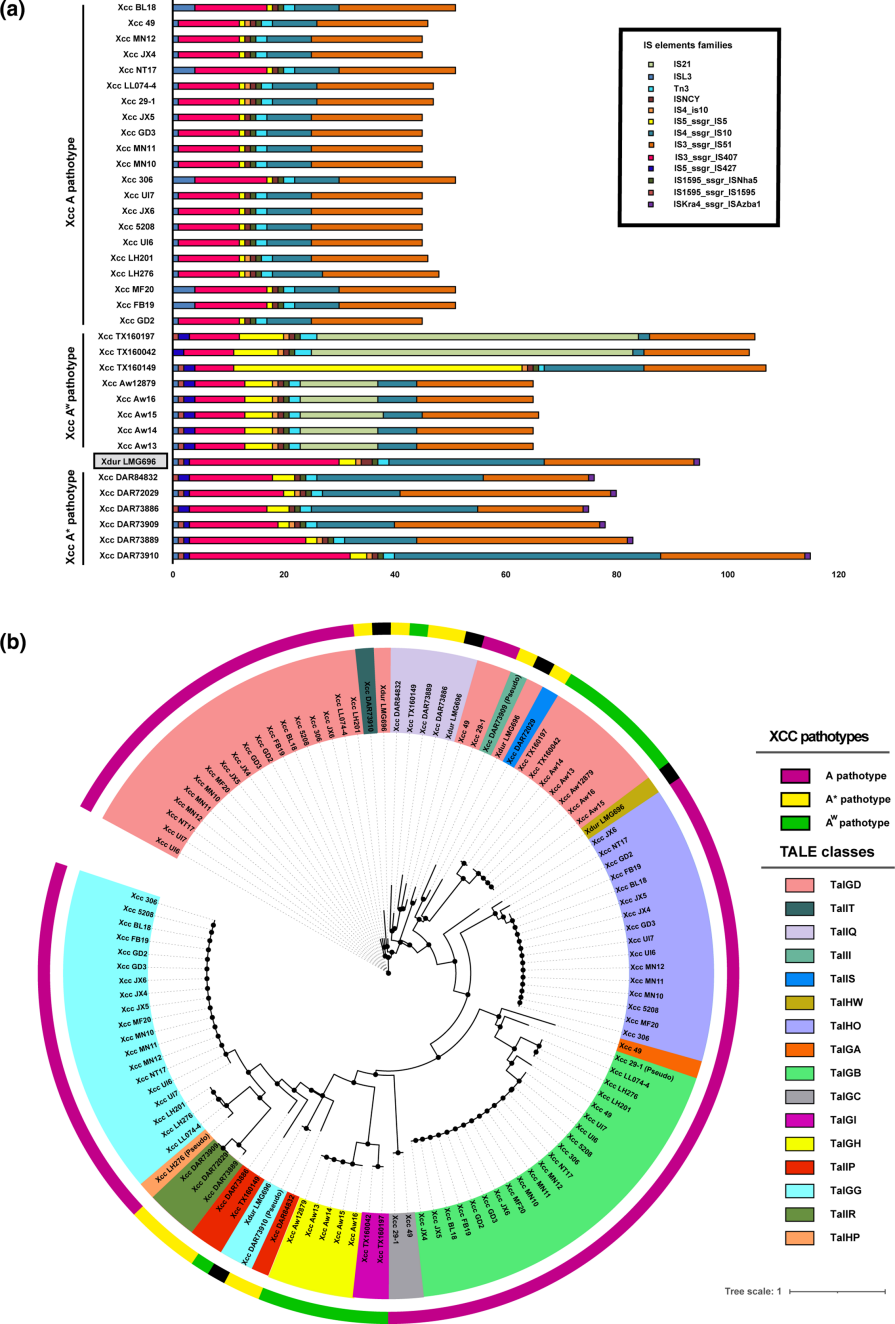
a) Distribution of IS elements in different IS element families (colour-coding given at the top right-hand side of image panel), where the x-axis represents numbers of IS elements in particular IS element families and the y-axis represents strains. b) A neighbor-joining phylogenetic tree of the central repeat region of TALEs constructed with DisTAL [32]. The outermost ring represents different Xcc pathotypes with Xdur LMG696 (black colour), and the innermost ring represents TALEs assigned to various classes with colour-coding given on the right-hand side of the panel.

The chromosomal sequence also encodes for a large number of IS elements indicating their role in the genome evolution of this pathogen. Xdur LMG696 harbours 95 IS elements which are in the range of A* pathotype IS elements, i.e. 75 to 115. In contrast, A pathotype strains carry a much smaller number of IS elements in the range of 45 to 51, and A^w^ pathotype has a variable number of IS elements from 65 to as much as 105 (Fig. 4a). A maximum number of IS elements fall into three IS element families, IS3_ssgr_IS51, IS3_ssgr_IS407, and IS4_ssgr_IS10. IS1595_ssgr_IS1595 and IS5_ssgr_IS427 families were present only in A* and A^w^ pathotypes, while IS21 was restricted to A^w^ pathotype group. ISKra4_ssgr_ISAzba1 family IS elements were present only in A* pathotypes and Xdur LMG696. Apart from these, IS elements from ISL3, IS5_ssgr_IS5, ISNCY, S1595_ssgr_ISNha5, IS4_is10, and Tn3 families were also present in varying numbers in all the strains (Fig. 4a). As mentioned above, XDLDRs were associated with IS elements, where XDLDR3 have a large number of IS elements belonging to various IS element families, IS3_ssgr_IS407, ISNCY, IS3_ssgr_IS51, IS5_ssgr_IS5, IS1595_ssgr_IS1595, and IS4_ssgr_IS10. IS elements were distributed throughout the Xdur LMG696 genome in large numbers, even outside the XDLDRs.

Transcription activator-like effectors (TALEs) are key virulence factors found in the *Xanthomonas* genus that manipulate host cell machinery for its own benefit. TALEs are tandem repeats of 33 to 34 amino acids, secreted by the type three secretion system into the host, where they act as transcription factors by binding to promotor elements using their repeat variable diresidues (RVDs) and thus regulating the expression of target genes [13, 34]. On account of being repetitive, TALEs are often missed in short-read sequencing technologies. However, the emergence of long-read third-generation sequencing technologies generating complete genome data has made it easier to study repetitive regions in depth. TALE analysis of Xdur revealed the presence of five TALEs distributed in four different classes (TalGD, TalGG, TalIQ, and TalHW), all encoded on the plasmids. The phylogenetic analysis of TALEs revealed a different repertoire of TALE classes in A*, A, and A^w^ pathotypes (Fig. 4b). As discussed in the above section Xdur was forming a clade with A* pathotypes, Xdur TALEs also grouped with TALEs of A* pathotypes, such as both TalGD and TalGG were clustered with TalIT and TalGG class of Xcc DAR73910, another TalGD was clustered with TalII of Xcc DAR73909, and TalIQ was clustered with Xcc DAR73889 and Xcc DAR73886. Xdur did not reveal any unrelated or new TALE class except for TalHW, which was confined only to Xdur. TalHW was associated with pLMG696-3 plasmid along with another TALE assigned to TalGD class.

## Conclusion

There are many reports focused on the most successful pathogens of the genus *Xanthomonas *. At the same time, some pathogens which infect more diverse plants, reported from the middle of the last century, are being neglected for their lower economic importance [7]. The present study reports the first high-quality complete genome sequence of a *Xanthomonas* pathovar that was reported from a diseased ornamental hedge plant [6]. Previous studies have hinted at its close relationship with *X. citri *, a citrus plant pathogen [9, 10]. Xdur LMG696 have four plasmids, two of which carry T4SS clusters similar to the ones found associated with plasmids of previously reported Xcc strains. Further, phylogenetic analysis of Xdur LMG696 with Xcc genomes revealed that Xdur LMG696 itself groups amongst Xcc A* strains, one of the Xcc pathotypes.

Comparative genomic analysis revealed regions unique to Xdur LMG696 with genes related to ICEs, prophages, and a large association of IS elements. The extensive prevalence of IS elements indicates chromosomal plasticity, and the association of unique regions with ICEs and phages suggests a role of horizontal gene transfer events. Mobile genetic elements (MGEs) such as plasmids, IS elements, ICEs and prophages are involved in genomic rearrangements and inter-strain variation, further contributing to the constant emergence of variable strains. Our report also focused on TALEs, which are considered important pathogenicity determinants in the genus *Xanthomonas *. TALE analysis revealed the presence of a unique TALE class in Xdur LMG696. These unique regions and TALEs might be good targets for molecular and pathogenicity studies.

## Supplementary Data

Supplementary material 1

## References

[1] Parte AC, Sardà Carbasse J, Meier-Kolthoff JP, Reimer LC, Göker M (2020). List of prokaryotic names with standing in nomenclature (LPSN) moves to the DSMZ. Int J Syst Evol Microbiol.

[2] Jacques M-A, Arlat M, Boulanger A, Boureau T, Carrère S (2016). Using ecology, physiology, and genomics to understand host specificity in *Xanthomonas*. Annu Rev Phytopathol.

[3] Ryan RP, Vorhölter F-J, Potnis N, Jones JB, Van Sluys M-A (2011). Pathogenomics of *Xanthomonas*: understanding bacterium-plant interactions. Nat Rev Microbiol.

[4] Timilsina S, Potnis N, Newberry EA, Liyanapathiranage P, Iruegas-Bocardo F (2020). *Xanthomonas* diversity, virulence and plant-pathogen interactions. Nat Rev Microbiol.

[5] NRCS U (2018). The PLANTS Database.

[6] Srinivasan M, Patel M (1957). Two new phytopathogenic bacteria on verbenaceous hosts. Curr Sci.

[7] Srinivasan MC, Patel MK, Thirumalachar MJ (1962). Two new phytopathogenic bacteria on verbenaceous hosts. Proceedings of the Indian Academy of Sciences - Section B.

[8] Gumtow RL, Khan AA, Bocsanczy AM, Yuen JMF, Palmateer AJ (2013). First report of a leaf spot disease of golden dewdrop (*Duranta erecta*) caused by *Pseudomonas cichorii* and a *Xanthomonas* species in Florida. Plant Dis.

[9] Bansal K, Midha S, Kumar S, Patil PB (2017). Ecological and evolutionary insights into *Xanthomonas citri* Pathovar diversity. Appl Environ Microbiol.

[10] Parkinson N, Cowie C, Heeney J, Stead D (2009). Phylogenetic structure of *Xanthomonas* determined by comparison of gyrB sequences. Int J Syst Evol Microbiol.

[11] Bansal K, Kumar S, Patil PB (2022). Phylo-taxonogenomics supports revision of taxonomic status of 20 *Xanthomonas* Pathovars to *Xanthomonas citri*. Phytopathology.

[12] Patané JSL, Martins J, Rangel LT, Belasque J, Digiampietri LA (2019). Origin and diversification of *Xanthomonas citri* subsp. *citri* pathotypes revealed by inclusive phylogenomic, dating, and biogeographic analyses. BMC Genomics.

[13] Boch J, Bonas U (2010). Xanthomonas AvrBs3 family-type III effectors: discovery and function. Annu Rev Phytopathol.

[14] Vandecraen J, Chandler M, Aertsen A, Van Houdt R (2017). The impact of insertion sequences on bacterial genome plasticity and adaptability. Crit Rev Microbiol.

[15] Lee H, Gurtowski J, Yoo S, Nattestad M, Marcus S (2016). Third-generation sequencing and the future of genomics. Bioinformatics.

[16] Wick RR, Judd LM, Gorrie CL, Holt KE (2017). Unicycler: resolving bacterial genome assemblies from short and long sequencing reads. PLoS Comput Biol.

[17] Walker BJ, Abeel T, Shea T, Priest M, Abouelliel A (2014). Pilon: an integrated tool for comprehensive microbial variant detection and genome assembly improvement. PLoS One.

[18] Parks DH, Imelfort M, Skennerton CT, Hugenholtz P, Tyson GW (2015). CheckM: assessing the quality of microbial genomes recovered from isolates, single cells, and metagenomes. Genome Res.

[19] Bushnell B, Egan R, Copeland A, Foster B, Clum A (2019). BBMap: a fast, accurate, splice-aware aligner. https://sourceforge.net/projects/bbmap.

[20] Grant JR, Arantes AS, Stothard P (2012). Comparing thousands of circular genomes using the CGView Comparison Tool. BMC Genomics.

[21] Stothard P, Wishart DS (2005). Circular genome visualization and exploration using CGView. Bioinformatics.

[22] Seemann T (2014). Prokka: rapid prokaryotic genome annotation. Bioinformatics.

[23] Page AJ, Cummins CA, Hunt M, Wong VK, Reuter S (2015). Roary: rapid large-scale prokaryote pan genome analysis. Bioinformatics.

[24] Guindon S, Dufayard J-F, Lefort V, Anisimova M, Hordijk W (2010). New algorithms and methods to estimate maximum-likelihood phylogenies: assessing the performance of PhyML 3.0. Syst Biol.

[25] Didelot X, Wilson DJ (2015). ClonalFrameML: efficient inference of recombination in whole bacterial genomes. PLoS Comput Biol.

[26] Letunic I, Bork P (2021). Interactive tree of life (iTOL) v5: an online tool for phylogenetic tree display and annotation. Nucleic Acids Res.

[27] Alikhan N-F, Petty NK, Ben Zakour NL, Beatson SA (2011). BLAST ring image generator (BRIG): simple prokaryote genome comparisons. BMC Genomics.

[28] Liu M, Li X, Xie Y, Bi D, Sun J (2019). ICEberg 2.0: an updated database of bacterial integrative and conjugative elements. Nucleic Acids Res.

[29] Arndt D, Grant JR, Marcu A, Sajed T, Pon A (2016). PHASTER: a better, faster version of the PHAST phage search tool. Nucleic Acids Res.

[30] Varani AM, Siguier P, Gourbeyre E, Charneau V, Chandler M (2011). ISsaga is an ensemble of web-based methods for high throughput identification and semi-automatic annotation of insertion sequences in prokaryotic genomes. Genome Biol.

[31] Grau J, Reschke M, Erkes A, Streubel J, Morgan RD (2016). AnnoTALE: bioinformatics tools for identification, annotation, and nomenclature of TALEs from *Xanthomonas* genomic sequences. Sci Rep.

[32] Pérez-Quintero AL, Lamy L, Gordon JL, Escalon A, Cunnac S (2015). QueTAL: a suite of tools to classify and compare TAL effectors functionally and phylogenetically. Front Plant Sci.

[33] Gordon JL, Lefeuvre P, Escalon A, Barbe V, Cruveiller S (2015). Comparative genomics of 43 strains of *Xanthomonas citri* pv. citri reveals the evolutionary events giving rise to pathotypes with different host ranges. BMC Genomics.

[34] Moscou MJ, Bogdanove AJ (2009). A simple cipher governs DNA recognition by TAL effectors. Science.

